# Co-regulation of Nr1d1 and Pparα in age-related changes of lipid metabolism and its modulation by calorie restriction

**DOI:** 10.18632/aging.206289

**Published:** 2025-07-28

**Authors:** Sang Gyun Noh, Hyun Woo Kim, Seungwoo Kim, Byung Pal Yu, Jeong-Hyun Yoon, Ki Wung Chung, Jaewon Lee, Hae Young Chung

**Affiliations:** 1 Research Institute for Drug Development, Pusan National University, Busan 46241, Republic of Korea; 2 Department of Pharmacy, College of Pharmacy, Pusan National University, Busan 46241, Republic of Korea; 3 Department of Physiology, The University of Texas Health Science Center at San Antonio, San Antonio, TX 78229, USA

**Keywords:** aging, calorie restriction, lipid metabolism, NR1D1, PPARα

## Abstract

Aging is associated with a decline in liver function, which increases the risk of age-related metabolic disorders. Calorie restriction (CR) counteracts age-related changes in the liver; however, the underlying molecular mechanism remains elusive. In this study, we integrated transcriptomic, bioinformatic, and molecular analyses to investigate the effects of aging and CR on age-related gene expression in the rat liver, focusing on the interplay between the circadian rhythm and lipid metabolism. Our results revealed aging-induced upregulation of *Nr1d1*, a key circadian repressor, and downregulation of *Ppara*, accompanied by decreased expression of fatty acid oxidation genes and increased expression of lipogenic genes. CR attenuated these age-related changes and restored circadian rhythm-related gene expression. Furthermore, we demonstrated that *Nr1d1* overexpression inhibited PPARα binding to peroxisome proliferator response elements (PPRE), resulting in decreased fatty acid oxidation gene expression. Our findings suggest that age-related dysregulation of *Nr1d1* contributes to impaired lipid metabolism in liver aging, and CR may exert its beneficial effects by modulating the interaction between NR1D1 and PPARα. This study provides novel insights into the molecular mechanisms linking circadian rhythms and lipid metabolism in hepatic aging.

## INTRODUCTION

Aging is a complex process characterized by progressive physical, functional, and physiological declines [1]. The liver, a vital organ responsible for maintaining homeostasis through various metabolic pathways, undergoes significant age-related morphological and functional changes with age [2]. These alterations can lead to the deterioration of liver function, increasing the risk of various diseases and negatively affecting overall health [3–6]. Therefore, efficient strategies to mitigate the effects of aging on the liver are crucial for promoting health and longevity.

Calorie restriction (CR), a well-established anti-aging intervention, has shown promise in modulating the aging process by regulating inflammation and metabolism [7, 8]. Several studies have reported beneficial effects of CR on liver metabolism and inflammation, with significant improvements in gene expression profiles and lipid accumulation associated with hepatic aging [9–15]. CR has been consistently recognized as the golden standard for anti-aging interventions, demonstrating robust effects on lifespan and health span across species. However, the precise mechanisms underlying the effects of CR on age-related changes in the liver remain unclear.

Recent research has highlighted the intricate interplay between metabolism and circadian rhythms. Circadian regulation of enzymes involved in metabolite synthesis, such as fatty acids and cholesterol, demonstrates the profound influence of circadian rhythms on metabolic gene expression [16, 17]. Genetic alterations in core clock genes can disrupt metabolic functions, leading to impaired gluconeogenesis, hepatic steatosis, obesity, and abnormal lipid and glucose metabolism [18–20]. Age-related declines in circadian rhythms have been linked to disruptions in metabolic tissue homeostasis [21–23], particularly in the liver [24–26]. Our previous study reported that aging induces dysregulation of circadian genes such as *Nr1d1*, suggesting a close relationship between the aging process and circadian clock dysfunction [27].

Lipid metabolism plays a central role in the aging process, with lipid accumulation exerting detrimental effects on cells and organs, contributing to age-related diseases and reduced lifespan [28–30]. Age-related changes in lipid composition and accumulation have been implicated in metabolic dysfunction-associated fatty liver disease (MAFLD), which increases in prevalence with age [31]. Lipid metabolism dysfunction accelerates the aging process [32], with several studies reporting increased hepatic lipid accumulation during aging [15, 33, 34]. Therefore, identifying key regulators linking circadian rhythms and lipid metabolism in aging contexts is crucial for developing effective healthy aging strategies.

Bioinformatics and systems biology approaches offer valuable tools for unraveling aging mechanisms and identifying critical aging biomarkers [35, 36]. These tools integrate statistical analyses, mathematical models, and pathway/network construction to provide comprehensive understanding of aging. Various studies using biological big data have shown metabolic changes during aging [37–41]. However, many bioinformatics studies remain at the systemic level and lack detailed exploration of specific mechanisms through which anti-aging interventions modulate aging. There is a pressing need for studies that bridge bioinformatics and systems biology with targeted biological experiments to elucidate the molecular underpinnings of aging and the effects of anti-aging interventions.

Our previous studies extensively characterized the beneficial effects of CR on various organs during aging [7, 14, 42]. Building on these findings, the study aimed to investigate age-related gene expression changes in the liver and identify key gene candidates modulated by CR. We employed an integrated approach combining RNA-seq analysis, bioinformatics tools, and targeted biological experiments. By focusing on metabolic homeostasis in the liver, we sought novel insights into molecular mechanisms underlying hepatic aging and the potential of CR to mitigate these effects. We hypothesized that CR counteracts age-related dysregulation of key circadian and metabolic regulators, thereby maintaining metabolic homeostasis in the aging liver. Through this investigation, we discovered that circadian and metabolic regulators Nr1d1 and Pparα were modulated by CR during aging.

## RESULTS

### Transcriptomic analysis of gene expression changes during aging and CR

**Figure 1:**
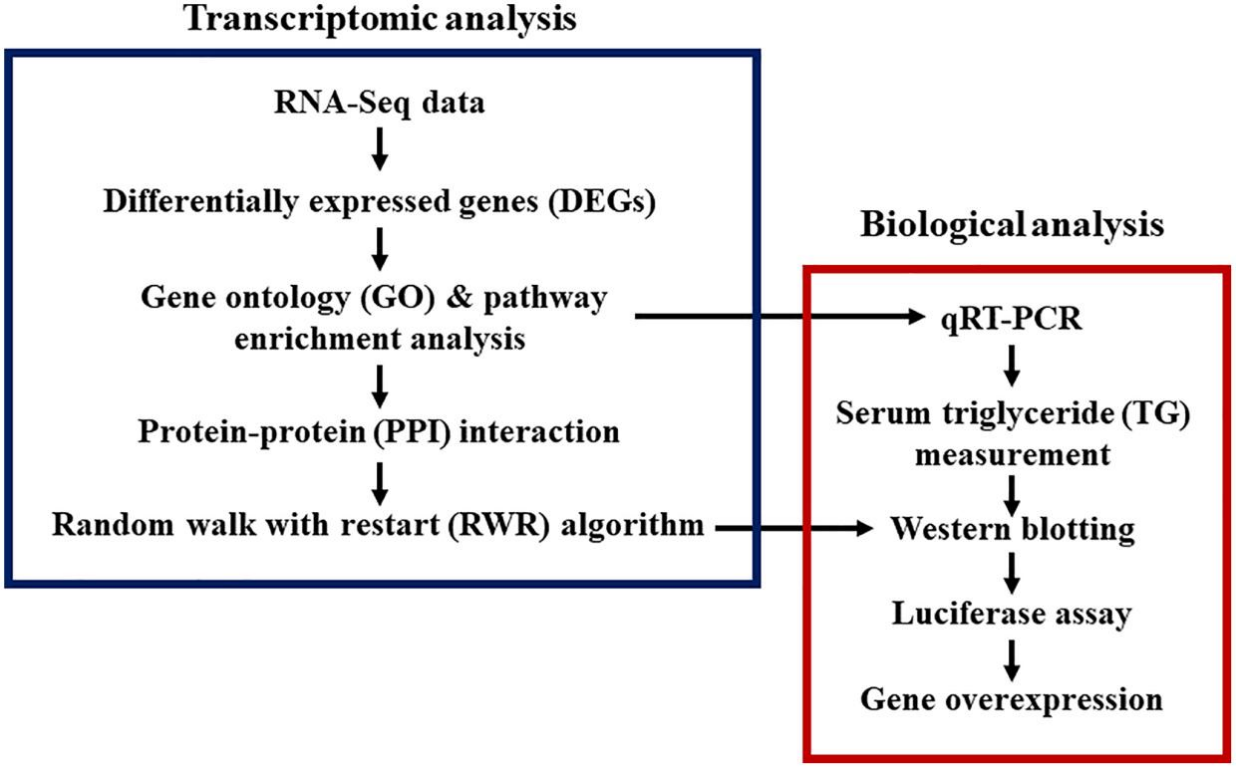
**Study designs for transcriptomic and biological analyses of aging and CR.** Bioinformatics, systems biology, and biological experiments were combined.

**Figure 2:**
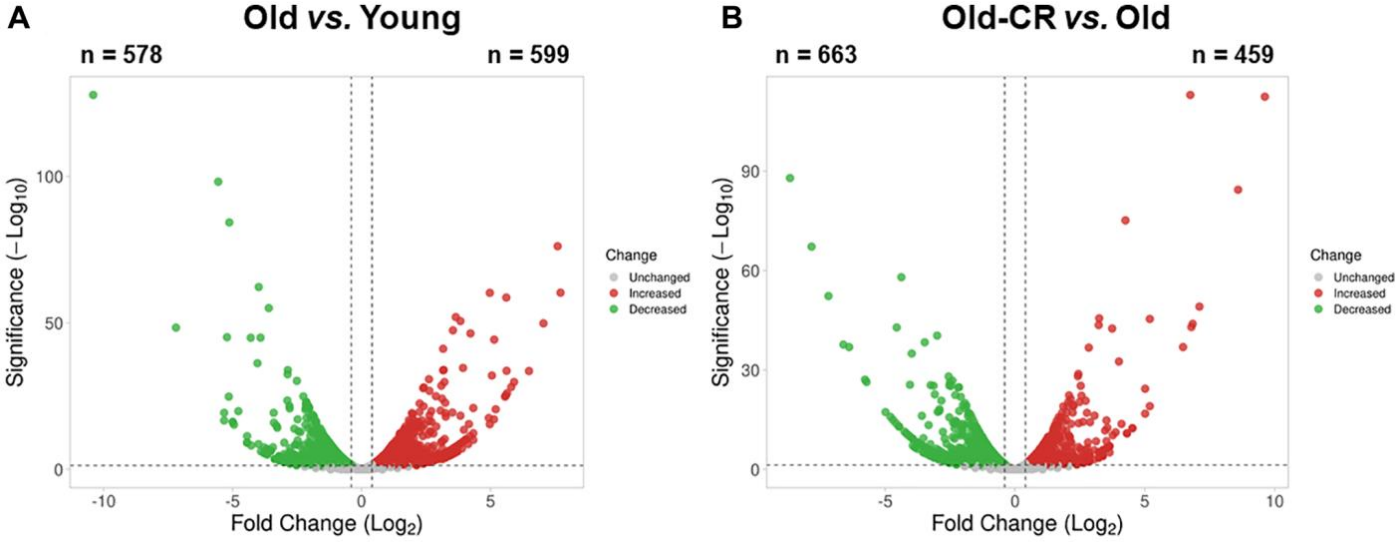
Aging-related differentially expressed genes from RNA-Seq data of the (**A**) Old vs. Young and (**B**) Old-CR vs. Old datasets from SD rats. Aging upregulated 599 genes and downregulated 578 genes, while CR in aged mice upregulated 459 genes and downregulated 663 genes. Red dots represent upregulated genes between the two groups, whereas green dots represent downregulated genes. Gray dots represent genes with no changes between the two groups. The DEG criteria were |FC|>1.5 and *p* < 0.05. Old-CR, old group under CR.

We developed an integrated approach combining bioinformatics, systems biology, and empirical biological analyses to investigate aging and CR effects on gene expression in the liver (Figure 1). Liver tissues from young rats (Young group), aged rats (Old group), and aged rats treated with CR (Old-CR group) were harvested for RNA-Seq analysis. We compared mRNA expression levels between the young and old groups (Old vs. Young) and between the old group and old-CR groups (Old-CR vs. Old) to identify differentially expressed genes (DEGs) (Supplementary Table 1). The Old vs. Young comparison revealed 599 upregulated genes and 578 downregulated genes (Figure 2A). In the Old-CR vs. Old comparison, 459 genes were upregulated, and 663 genes were downregulated (Figure 2B).

To elucidate DEG functions, we analyzed all genes using the Database for Annotation, Visualization, and Integrated Discovery (DAVID) across five categories: biological processes (BP), cellular components (CC), molecular functions (MF), Kyoto Encyclopedia of Genes and Genomes (KEGG), and Reactome. The top 30 terms based on *p*-values are listed, to provide insights into the gene functions affected by aging and CR (Supplementary Table 2).

**Figure 3:**
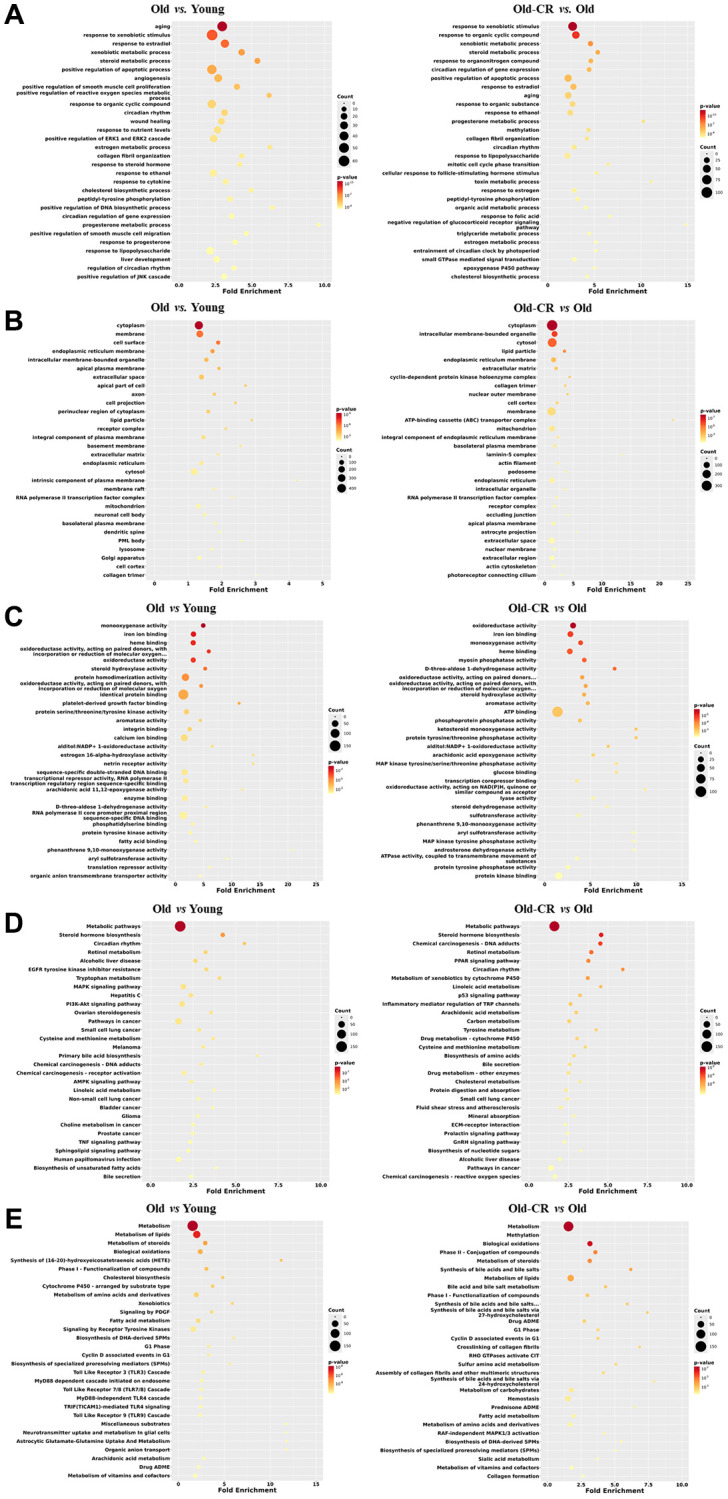
**Top 30 enriched GO or pathway terms of the DEGs in the Old vs. Young and Old-CR vs. Old datasets from SD rats.** (**A**) BP, (**B**) CC, (**C**) MF, (**D**) KEGG, and (**E**) Reactome data. Gene ontology analysis revealed significant alterations in metabolic processes, circadian rhythm, and inflammation (BP); lipid particles, collagen, and mitochondria (CC); and energy/lipid metabolism enzymes (MF). Pathway analysis identified changes in AMPK/PPAR signaling and circadian rhythm (KEGG), with distinct inflammatory signatures in aging and extracellular matrix responses in CR (Reactome). The criterion for a significant term was *p* < 0.05. Counts indicate the number of genes related to each term in the analysis. The fold enrichment indicates how much a particular GO term or pathway is overrepresented in the list of genes compared to that expected by chance. Old-CR, old group under CR.

Our results revealed significant changes across various metabolic processes in both Old vs. Young and Old-CR vs. Old datasets. Prevalent BP terms included “aging,” “steroid metabolic process,” “triglyceride metabolic process,” and “xenobiotic metabolic process” (Figure 3A). Key circadian rhythm terms included “circadian rhythm” and “regulation of circadian rhythm,” while immune response terms included “response to lipopolysaccharide”.

CC terms such as “lipid particle,” “collagen trimer,” and “mitochondrion,” were altered in both datasets (Figure 3B). Key MF terms associated with energy metabolism-related “oxidoreductase” and “monooxygenase” and lipid metabolism-related “steroid hydroxylase activity” and “fatty acid binding” (Figure 3C).

KEGG analysis revealed changes in “AMPK signaling pathway,” “PPAR (Peroxisome proliferator-activated receptors) signaling pathway,” and “steroid hormone biosynthesis,” indicating strong associations with lipid metabolism and circadian rhythm-related terms were also identified (Figure 3D). Reactome terms such as “metabolism,” “metabolism of lipids/steroids,” and “fatty acid metabolism,” were prominent in both datasets. The Old vs. Young dataset showed a higher prevalence of terms related to toll-like receptor signaling, whereas the Old-CR vs. Old dataset was enriched in terms related to the extracellular matrix (ECM) terms, such as “crosslinking of collagen fibrils” and “collagen formation” (Figure 3E).

These results suggest that genes associated with inflammation, metabolism, circadian rhythms, and the ECM exhibited differential expression patterns in aging and CR. Consistent changes in metabolism and circadian rhythm-related GOs and pathways highlight their significant interplay in the aging liver, underscoring their critical roles in liver responses to aging and CR interventions.

### CR modulation of aging-related changes in lipid metabolism-related gene expression

To explore CR intervention in age-induced changes, we examined DEG expression patterns from both datasets (Supplementary Table 3). Specifically, we conducted GO and pathway analyses focusing on genes upregulated in the Old vs. Young but downregulated in the Old-CR vs. Old (Table 1). BP terms related to fatty acid, lipid, and cholesterol steroid processes and MF terms, including palmitoyl-CoA 9-desaturase and stearoyl-CoA 9-desaturase activities were identified. Metabolic pathways such as the AMPK and PPAR signaling pathways, lipid metabolism, and fatty acid metabolism were altered.

**Table 1:** GO terms and pathways of genes that were upregulated in the Old vs. Young but downregulated in the Old-CR vs. Old datasets.

**Category**	**Term**	**Fold enrichment**	***p*-value**	**Genes**
BP	positive regulation of apoptotic process	4.8095	1.60E-05	MOAP1, TNFRSF12A, SRC, ZBTB16, PDCD5, PTGS2, PPP2CA, CASP12, UBD, ALDH1A1, HMOX1, TSPO, CYP1B1
	fatty acid biosynthetic process	16.37276596	2.37E-04	ACLY, SCD, FASN, SCD2, ACACB
	response to organonitrogen compound	10.68777778	0.001206086	CASP12, UBD, CCNG1, PTGS2, AACS
	acetyl-CoA metabolic process	51.30133333	0.001440356	ACLY, FASN, ACACB
	peptidyl-tyrosine phosphorylation	9.271325301	0.002040379	EFEMP1, SPINK1, SRC, PDGFB, EPHA3
	response to xenobiotic stimulus	3.129286506	0.002650987	CASP12, SRC, ALDH1A1, PDGFB, HMOX1, TSPO, MVD, CYP4A2, PTGS2, ACACB, AACS
	response to fatty acid	13.38295652	0.003241543	SCD, SRC, SCD2, PTGS2
	regulation of cell-cell adhesion	28.857	0.00466156	SRC, ADAM8, ZDHHC2
	response to organic cyclic compound	3.800098765	0.005063243	G6PD, LUM, ALDH1A1, PDGFB, CYP1B1, PTGS2, ACACB, AACS
	positive regulation of ERK1 and ERK2 cascade	4.397257143	0.005141411	CCL21, SRC, C5AR2, TRPV4, PDGFB, GAS6, FGF21
	inflammatory response	3.439195531	0.008574019	CXCL9, CCL21, ELF3, C5AR2, FASN, ADAM8, CXCL1, PTGS2
	negative regulation of cell proliferation	3.01772549	0.009810343	ZBTB16, PDCD5, ADORA1, CYP1B1, HMOX1, CD9, CDH13, SLIT3, PTGS2
	lipid biosynthetic process	19.238	0.010367306	ACLY, SCD, FASN
	white fat cell differentiation	19.238	0.010367306	SCD, PNPLA3, AACS
	positive regulation of protein kinase B signaling	5.785864662	0.010820854	CCL21, SRC, GDF15, ADAM8, GAS6
	positive regulation of DNA biosynthetic process	17.75815385	0.012106014	SRC, PDGFB, CYP1B1
	neutrophil chemotaxis	8.208213333	0.012599584	LGALS3, CXCL9, CCL21, CXCL1
	positive regulation of phosphatidylinositol 3-kinase activity	15.3904	0.015934563	CCL21, SRC, PDGFB
	activation of protein kinase B activity	15.3904	0.015934563	SRC, PDGFB, GAS6
	positive regulation of MAP kinase activity	7.417060241	0.016521383	SRC, PDGFB, ADAM8, TPD52L1
	response to ethanol	4.014886957	0.016714469	G6PD, SPINK1, CASP12, GSN, ALDH1A1, AACS
	cell chemotaxis	7.242541176	0.017596824	CCL21, ARHGEF16, PDGFB, CXCL1
	monocyte chemotaxis	14.4285	0.018017922	LGALS3, CCL21, PDGFB
	endothelial cell-cell adhesion	102.6026667	0.019213353	CYP1B1, THBS4
	positive regulation of smooth muscle cell proliferation	6.917033708	0.019862531	PDGFB, HMOX1, CDH13, PTGS2
	positive regulation of protein tyrosine kinase activity	13.57976471	0.020209789	TRPV4, PDGFB, GAS6
	actin filament organization	4.607904192	0.023008755	GSN, TRPV4, MYO7B, RHOC, TLE6
	positive regulation of reactive oxygen species metabolic process	12.4787027	0.023694306	PDGFB, CYP1B1, TSPO
	aging	3.131767442	0.024104355	CDKN1C, CASP12, GSN, CNP, TSPO, PTGS2, DCN
	antimicrobial humoral immune response mediated by antimicrobial peptide	6.346556701	0.024854734	LGALS3, CXCL9, CCL21, CXCL1
	positive regulation of glomerular filtration	76.952	0.025536074	PDGFB, GAS6
	monounsaturated fatty acid biosynthetic process	76.952	0.025536074	SCD, SCD2
	adrenal gland development	11.5428	0.027405876	CDKN1C, CYP1B1, TSPO
	positive regulation of JNK cascade	6.095207921	0.027581281	CCL21, TRPV4, TPD52L1, ANKRD6
	immune system process	11.26126829	0.028691809	IGHM, IRF7
	cholesterol biosynthetic process	10.99314286	0.030001514	G6PD, MVD, LSS
	positive regulation of inflammatory response	5.863009524	0.030460944	TRPV4, NKG7, ADAM8, IL17RB
	fructose catabolic process	61.5616	0.031818362	ALDH1A1, ALDH1A7
	positive regulation of smooth muscle cell migration	10.26026667	0.03406973	SRC, PDGFB, CYP1B1
	response to nutrient levels	4.071534392	0.034078992	SPINK1, SRC, FASN, ACACB, FGF21
	cell adhesion	2.88055615	0.034278073	TNFRSF12A, SRC, CYP1B1, CD9, CCDC141, MPDZ, THBS4
	positive regulation of I-kappaB kinase/NF-kappaB signaling	3.966597938	0.036959971	CCL21, UBD, HMOX1, RHOC, TMEM106A
	response to iron (III) ion	51.30133333	0.038060473	G6PD, CASP12
	kidney development	3.926122449	0.038150589	CDKN1C, ALDH1A1, RHPN1, CYP4A2, DCN
	negative regulation of dendritic cell apoptotic process	43.97257143	0.044262663	CCL21, GAS6
	cellular response to glucose stimulus	4.88584127	0.048049626	PPP2CA, GAS6, AACS, FGF21
	chemokine-mediated signaling pathway	8.394763636	0.049034302	CXCL9, CCL21, CXCL1
CC	extracellular space	2.208098626	5.51E-04	WFDC21, CXCL9, SPINK1, GSN, CCL21, CNP, GDF15, LUM, PDGFB, MUP4, CTSW, CXCL1, PLA2G7, THBS4, DCN, LGALS3, PROCR, EFEMP1, PODNL1, CDH13, SLIT3, GAS6, FGF21
	cytoplasm	1.393257601	0.00602873	CDKN1C, LGALS3, EFEMP1, CASP12, ME1, ANXA8, CLIC2, SULT4A1, HTATIP2, ANKRD6, CDKL2, G6PD, SPINK1, MYO7B, DUSP26, IL17RB, TLE6, ACLY, EVC, ALDH1A1, IRF7, CDH13, ADAM8, GAS6, CNP, SRC, PDGFB, DCUN1D2, PTGS2, PLA2G7, PPP2CA, UBD, CCDC141, MPDZ, PDLIM4, FGF21, NEK8, MOAP1, GSN, GDF15, MOK, PRSS36, TPD52L1, KY, SBK1, FASN, CCNG1, PNPLA3, B9D1, PNPLA5
	caveola	6.761153054	0.02109186	SRC, HMOX1, CDH13, PTGS2
	podosome	13.2405914	0.0212081	GSN, SRC, ADAM8
	membrane	1.41390075	0.033419567	POPDC2, ADAMDEC1, CNP, SRC, C5AR2, PDGFB, CYP4A2, SLC2A5, ZDHHC2, ADGRG2, SCD2, HMOX1, CYP1B1, SLIT3, TAS1R1, CCDC141, CLIC2, CSMD1, GRIA3, ENTPD7, SYT15, RHOC, IL17RB, PROCR, SCD, TRPV4, PNPLA3, CD9, PNPLA5, B9D1, PLP2, TMEM106A
	extracellular region	1.943573049	0.043080377	LGALS3, WFDC21, GSN, GDF15, PDGFB, CD9, PRSS36, GAS6, ZP2, CHRDL1, DCN, THBS4
MF	growth factor activity	7.79222973	2.71E-04	EFEMP1, GDF15, PDGFB, CXCL1, GAS6, THBS4, FGF21
	protein homodimerization activity	2.678861789	0.004834941	G6PD, GDF15, ZBTB16, PDGFB, CRYL1, HMOX1, CDH13, MVD, TPD52L1, PTGS2, PDLIM4, ZDHHC2
	collagen binding	9.152777778	0.009337216	LUM, PDGFB, DCN, THBS4
	chemokine activity	13.35810811	0.020808064	CXCL9, CCL21, CXCL1
	benzaldehyde dehydrogenase (NAD+) activity	82.375	0.023840283	ALDH1A1, ALDH1A7
	palmitoyl-CoA 9-desaturase activity	82.375	0.023840283	SCD, SCD2
	heme binding	4.476902174	0.025071838	SRC, CYP1B1, HMOX1, CYP4A2, PTGS2
	stearoyl-CoA 9-desaturase activity	65.9	0.029711838	SCD, SCD2
	protein binding	1.694571429	0.03195988	CDKN1C, UNC5B, SRC, ZBTB16, PTGS2, PPP2CA, EFEMP1, CASP12, TRPV4, FASN, ADORA1, HMOX1, TSPO, CD9, CCDC141, PDLIM4, MPDZ, GRIA3
	3-chloroallyl aldehyde dehydrogenase activity	54.91666667	0.035548404	ALDH1A1, ALDH1A7
	glyceraldehyde-3-phosphate dehydrogenase (NAD+) (non-phosphorylating) activity	47.07142857	0.041350187	ALDH1A1, ALDH1A7
KEGG	AMPK signaling pathway	5.919354839	0.009421368	PPP2CA, SCD, FASN, SCD2, ACACB
	Alcoholic liver disease	5.48088411	0.012249191	SCD, FASN, SCD2, CXCL1, ACACB
	Metabolic pathways	1.749653733	0.014414787	MT-ND6, G6PD, CYP4A2, PTGS2, ACACB, LSS, PLA2G7, AACS, ACLY, SCD, FASN, ALDH1A1, ME1, SCD2, PNPLA3, CRYL1, HMOX1, MVD, ALDH1A7
	PPAR signaling pathway	6.882970743	0.019366476	SCD, SCD2, ME1, CYP4A2
	Cytokine-cytokine receptor interaction	3.276395667	0.03364291	CXCL9, TNFRSF12A, CCL21, GDF15, CXCL1, IL17RB
Reactome	Metabolism of lipids	2.817831233	0.004103554	ACLY, SCD, FASN, PNPLA3, CYP1B1, TSPO, PNPLA5, MVD, PTGS2, LSS, AACS
	G alpha (i) signalling events	3.685106717	0.010239663	PPP2CA, CXCL9, CCL21, SRC, ADORA1, CXCL1, TAS1R1
	GPCR downstream signalling	2.649684044	0.016729272	PPP2CA, CXCL9, CCL21, SRC, ARHGEF16, ADORA1, CXCL1, TAS1R1, RHOC
	Metabolism	1.670611517	0.019928616	MT-ND6, G6PD, LUM, PTGS2, LSS, AACS, DCN, ACLY, SCD, FASN, ALDH1A1, PNPLA3, CRYL1, HMOX1, TSPO, CYP1B1, PNPLA5, MVD, SULT4A1
	Fatty acyl-CoA biosynthesis	12.85249042	0.021695752	ACLY, SCD, FASN
	Fatty acid metabolism	4.501476114	0.023057567	ACLY, SCD, FASN, CYP1B1, PTGS2
	G1 Phase	9.808479532	0.035914728	PPP2CA, CDKN1C, SRC
	Cyclin D associated events in G1	9.808479532	0.035914728	PPP2CA, CDKN1C, SRC
	Chemokine receptors bind chemokines	8.470959596	0.046907572	CXCL9, CCL21, CXCL1
	Signaling by GPCR	2.162798195	0.048288983	PPP2CA, CXCL9, CCL21, SRC, ARHGEF16, ADORA1, CXCL1, TAS1R1, RHOC

**Figure 4:**
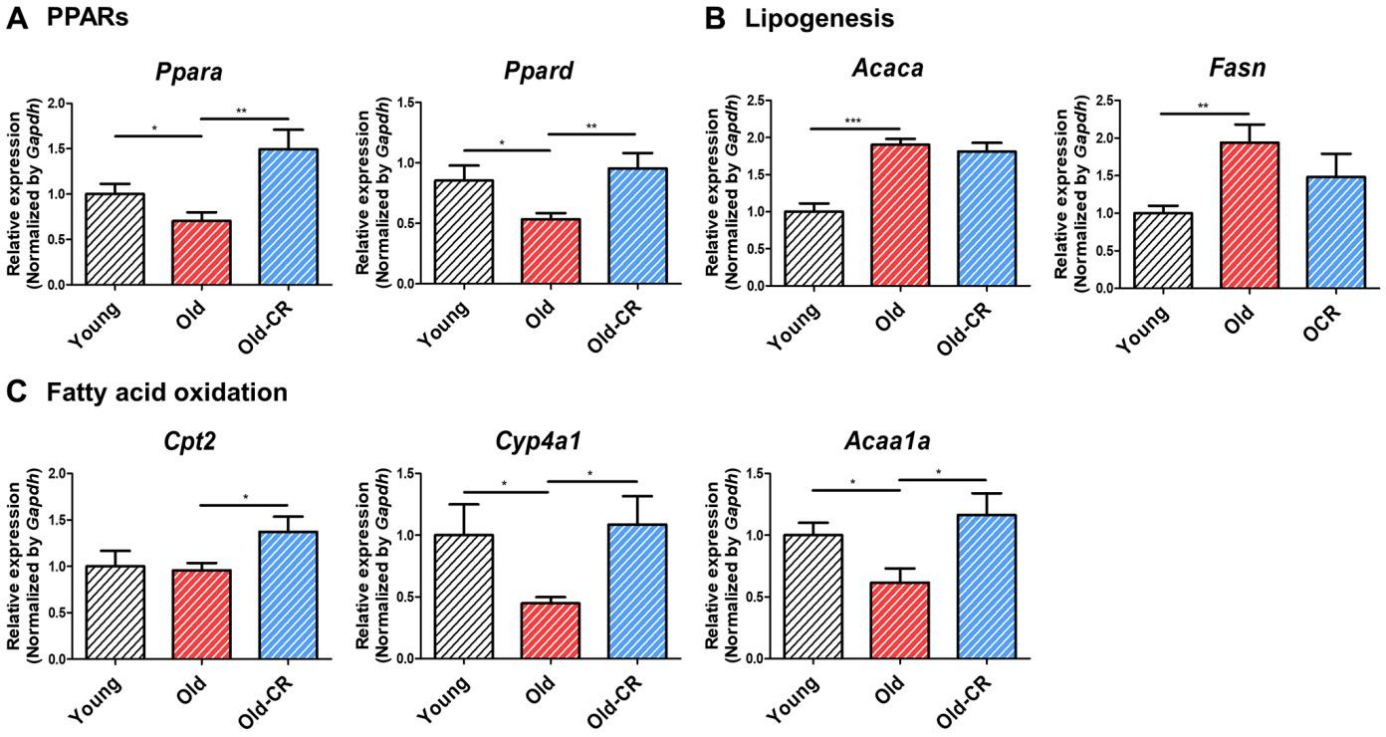
**Relative mRNA expression of genes related to lipid metabolism that are differentially expressed during aging and CR (*n* = 6 per group).** (**A**) *Ppara* and *Ppard* are downregulated in aging but upregulated in CR, (**B**) while the lipogenic genes *Acaca* and *Fasn* display opposite trends. (**C**) Fatty acid oxidation-related genes, such as *Cpt2*, *Cyp4a1*, and *Acca1a*, are downregulated in aging but upregulated in CR. Data are presented as mean ± SEM. ^*^p < 0.05, ^**^p < 0.01, and ^***^p < 0.001 between two groups.

Examination of lipogenesis and fatty acid oxidation genes revealed that lipogenic genes (*Acly, Scd*, *Fasn*) increased with aging and decreased with CR, while fatty acid oxidation genes showed opposite trends (Table 2). qRT-PCR was validated that PPARs and fatty acid oxidation genes (*Ppara, Ppard, Cpt2, Cyp4a1, Acaa1a*) were downregulated during aging but upregulated with CR, whereas the lipogenic genes (*Acaca*, *Fasn*) showed the opposite results (Supplementary Table 4 and Figure 4).

**Table 2:** Changes in the expression of genes related to lipid metabolism from RNA-Seq data in the Old vs. Young and Old-CR vs. Old datasets.

**Category**	**Genez**	**Old vs. Young**	***p*-value**	**Old-CR vs. Old**	***p*-value**
Lipogenesis	*Aacs*	3.972369982	3.30E-08	−2.02791896	2.00E-03
	*Acly*	1.777685362	9.50E-03	−1.729074463	1.10E-02
	*Acaca*	1.945309895	4.90E-03	1.049716684	8.30E-01
	*Acacb*	1.558329159	4.90E-02	−1.905275996	3.00E-03
	*Fasn*	2.203810232	4.20E-04	−2.056227653	8.90E-04
	*Pnpla3*	6.105036836	4.30E-14	−2.887858391	1.80E-06
	*Pparg*	−1.197478705	6.00E-01	−1.148698355	7.40E-01
	*Scd*	1.777685362	9.80E-03	−4.469148552	1.70E-11
	*Scd2*	3.052518418	3.30E-05	−2.099433367	2.90E-03
Fatty acid oxidation	*Acox1*	−1.125058485	5.90E-01	1.494849249	6.30E-02
	*Cpt1a*	−1.021012126	9.20E-01	1.433955248	9.20E-02
	*Cpt2*	1.205807828	4.00E-01	1.283425898	2.50E-01
	*Acadvl*	−1.079228237	7.20E-01	1.042465761	8.50E-01
	*Hadhb*	1.222640278	3.70E-01	1.248330549	3.10E-01
	*Acadm*	−1.172834949	4.70E-01	−1.117287138	6.00E-01
	*Echs1*	−1.515716567	6.30E-02	1.292352831	2.40E-01
	*Hadh*	−1.125058485	6.00E-01	−1.189207115	4.30E-01
	*Ppara*	−1.164733586	5.70E-01	1.394743666	1.70E-01
	*Ppard*	−2.531513188	2.40E-04	1.310393404	2.90E-01
	*Acox1*	−1.125058485	5.90E-01	1.494849249	6.30E-02

**Figure 5:**
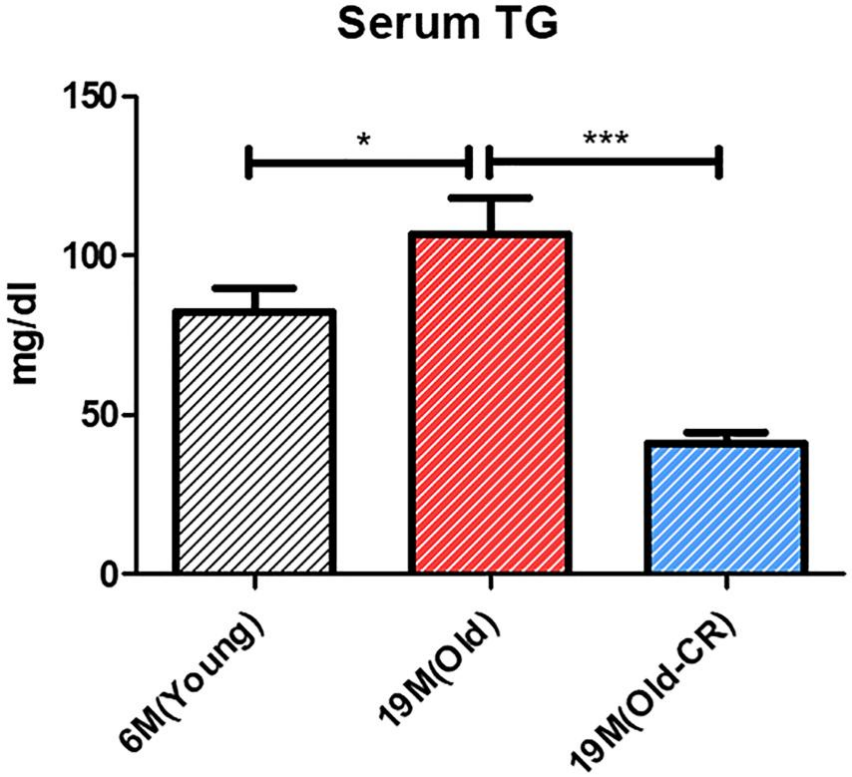
**Effect of aging and CR on serum TG (*n* = 6 per group).** Serum TG levels are increased in Old rats compared to those in Young rats and are decreased in Old-CR rats compared to those in Old rats (Young: 82.28 ± 7.111 mg/dL, Old: 106.8 ± 11.23 mg/dL, and Old-CR: 40.73 ± 3.689 mg/dL). Data are presented as mean ± SEM. Old-CR, old group under CR; TG, triglycerides.

Triglyceride (TG) levels were measured to assess the aging and CR effects on lipid metabolism. TG levels were significantly elevated in the Old group compared to Young (106.8 ± 11.23 vs. 82.28 ± 7.111 mg/dL; *p* < 0.05); but significantly reduced in the Old-CR compared to Old (40.73 ± 3.689 mg/dL; *p* < 0.0001) (Figure 5). These findings indicate that lipid synthesis genes increased with aging and decreased with CR, affecting lipid accumulation and suggesting that changes in lipogenesis and fatty acid oxidation are crucial in hepatic aging, with CR potentially counteracting these alterations.

### Expression changes of the circadian gene Nr1d1 predicted as a key gene linking circadian rhythms and metabolism by CR during aging

**Figure 6:**
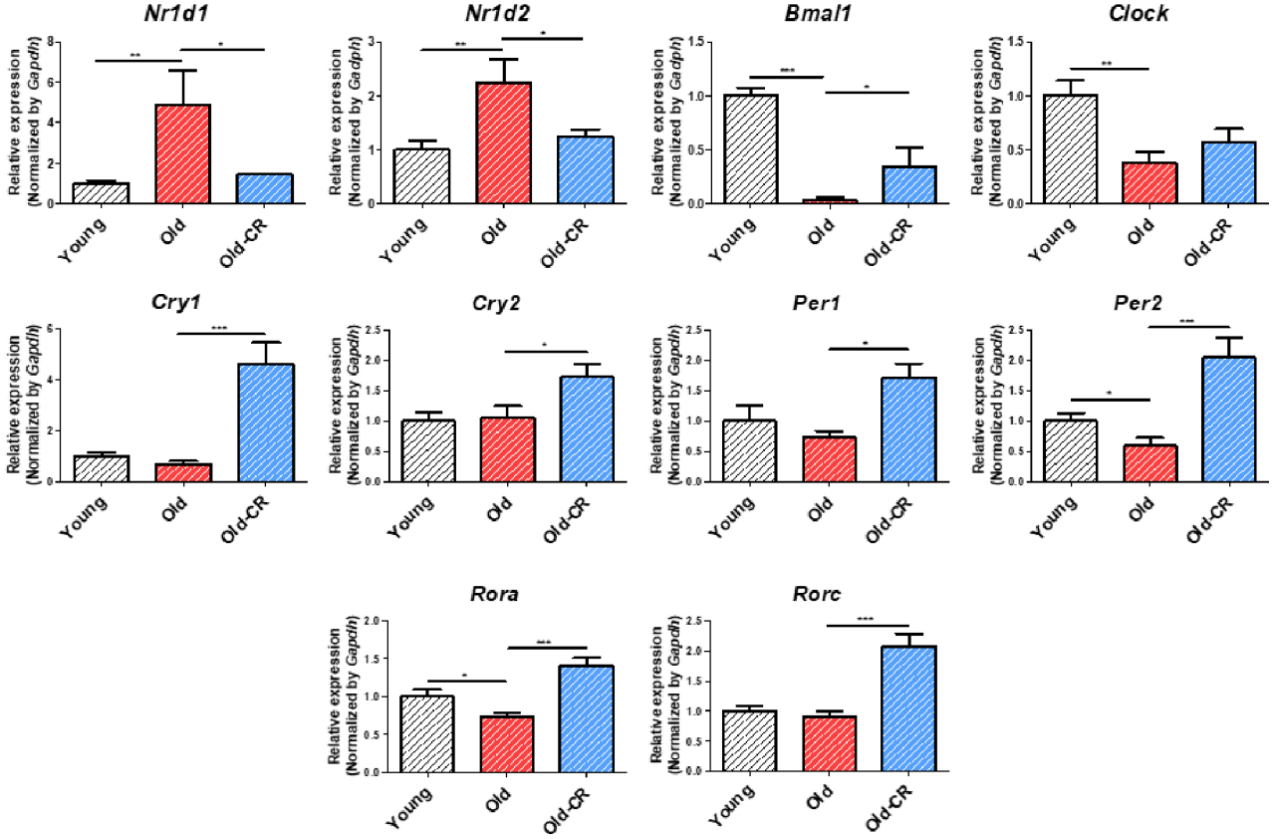
**Relative mRNA expression of circadian genes that are differentially expressed during aging and CR (*n* = 6 per group).*** Nr1d1* and *Nr1d2* are upregulated in aging but downregulated in CR, while *Bmal1*, *Per2*, and *Rora* display opposite trends. *Clock* is downregulated in aging. *Cry1*, *Cry2*, *Per1*, and *Rorc* are upregulated in CR. Data are presented as mean ± SEM. ^*^p < 0.05, ^**^p < 0.01, and ^***^p < 0.001 between two groups.

Following lipid metabolism analysis, we examined circadian rhythm genes to identify aging- and CR-related expression changes (Table 3). Most circadian genes showed significant alterations in both Old vs. Young and Old-CR vs. Old datasets, with *Cry1*, *Cry2*, *Nfil3*, *Nr1d1*, and *Rorc* displaying opposite trends in both datasets. We focused on *Nr1d1*, a circadian repressor that increased with age. Furthermore, qRT-PCR validated circadian gene changes (Figure 6 and Supplementary Table 4), showing that *Nr1d1* and *Nr1d2* increased in the Old group compared to Young and decreased in the Old-CR compared to Old. Conversely, *Bmal1* decreased with aging but increased with CR. *Clock* also decreased with aging. Other genes such as *Rora*, *Rorc*, *Per1*, *Per2*, *Cry1*, and *Cry2* showed with aging and significant increases with CR.

**Table 3:** Changes in the expression of circadian genes from RNA-Seq data in the Old vs. Young and Old-CR vs. Old datasets.

	**Old vs. Young**	***p*-value**	**Old-CR vs. Old**	***p*-value**
*Bmal1* (*Arntl*)	−3.116658319	4.20E-06	−9.51365692	9.20E-12
*Clock*	−1.404444876	1.30E-01	−1.879045498	5.40E-03
*Cry1*	−1.777685362	4.80E-02	4.75682846	4.90E-09
*Cry2*	−1.021012126	9.30E-01	2.099433367	1.00E-03
*Dbp*	12.55334557	2.60E-23	1.945309895	2.10E-03
*Nfil3*	−1.647182035	3.10E-02	1.569168196	4.70E-02
*Npas2*	−2.848100391	1.20E-05	−23.58830748	2.50E-19
*Nr1d1*	2.514026749	5.30E-05	−1.214194884	3.80E-01
*Nr1d2*	1.591072968	4.00E-02	1.484523571	7.00E-02
*Per1*	1.021012126	9.20E-01	2.281527432	2.20E-04
*Per2*	1.753211443	1.60E-02	4.228072162	2.00E-10
*Rora*	−1.292352831	6.70E-01	1.2397077	7.80E-01
*Rorb*	1	1.00E+00	1	1.00E+00
*Rorc*	−1.01395948	9.60E-01	2.0139111	1.50E-03

**Figure 7:**
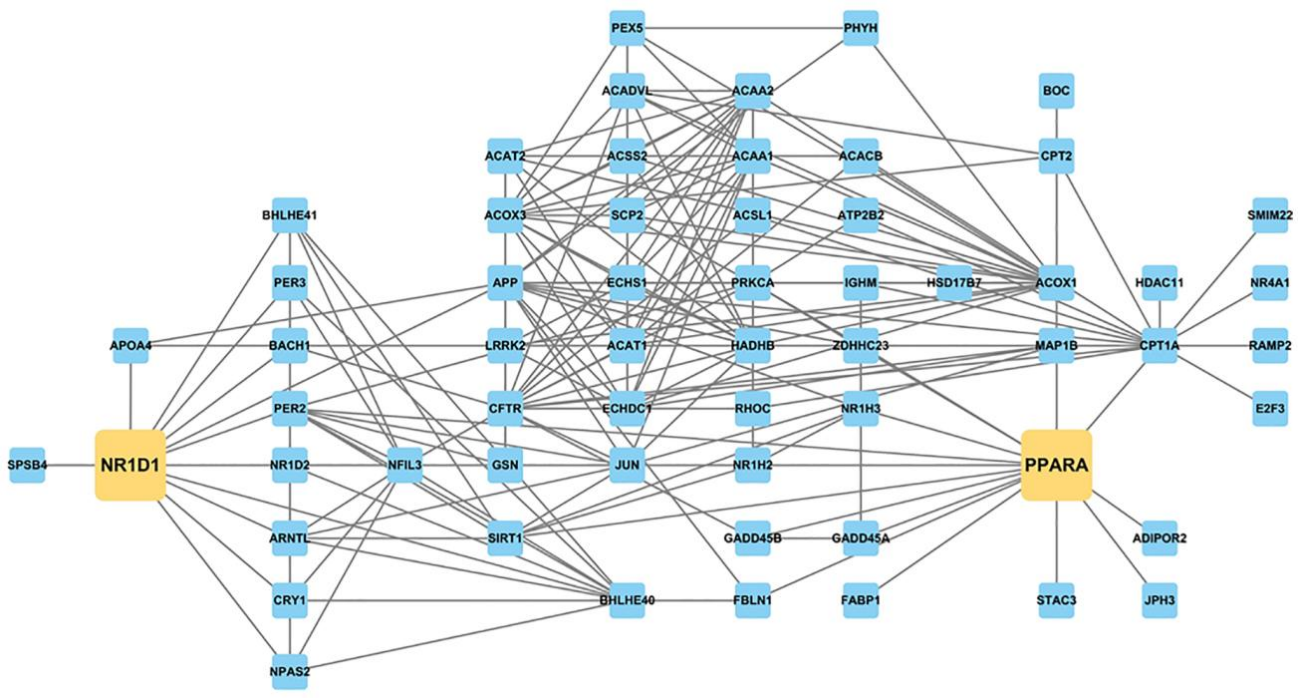
PPI network constructed with genes related to circadian rhythm and lipid metabolism and aging-altered DEGs, focusing on *Nr1d1* and *Ppara.* NR1D1 is highly related to several circadian and metabolic regulators, such as *Bmal1 (Arntl)*, *Nr1d2*, *Cry1*, *Acox1*, *Cpt2*, and *Ppara*.

We constructed a protein-protein interaction (PPI) network based on circadian-associated genes, lipid metabolism-related genes, and aging-altered DEGs, trimmed to focus on *Nr1d1* (Supplementary Figure 1 and Figure 7, Supplementary Table 5). Results confirmed that circadian rhythm and lipid metabolism genes were interconnected around *Nr1d1*, with *Ppara* among these connections. These analyses suggest that increased *Nr1d1* expression during aging affects both circadian rhythm and lipid metabolism, which was ameliorated by CR.

### Co-regulation of Nr1d1- and Pparα-induced age-related changes in lipid metabolism and its modulation by CR

To identify the genes closely associated with *Nr1d1*, we applied the RWR algorithm using *Nr1d1* as the seed node (Supplementary Figure 1 and Supplementary Table 6). Among the top 100 genes, several circadian and lipid metabolism genes were identified, such as *Cry1*, *Arntl*, *Nr1d2*, *Fasn*, *Pparg*, and *Acaca*. *Ppara* ranked among top genes, indicating that Nr1d1 is closely associated with Pparα, a major metabolic regulator.

**Figure 8:**
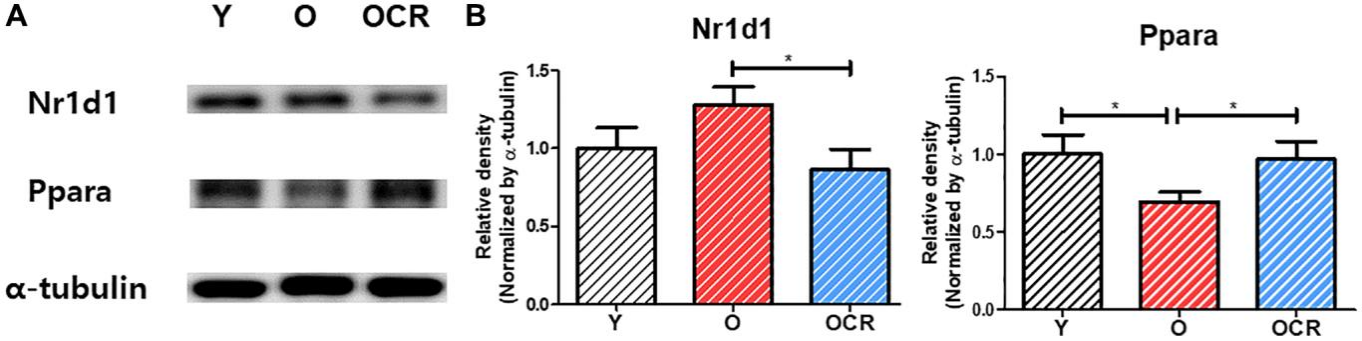
**Protein expression of NR1D1 and PPARα during aging and CR (*n* = 6 per group).** (**A**) Representative images of NR1D1 and PPARα bands. (**B**) Relative expression of NR1D1 and PPARα. The expression of NR1D1 increases during aging and decreases under CR. In contrast, the expression of PPARα is downregulated during aging but upregulated by CR. Data are presented as mean ± SEM. Abbreviations: Y: Young; O: Old; OCR: Old-CR. ^*^p < 0.05 between two groups.

**Figure 9:**
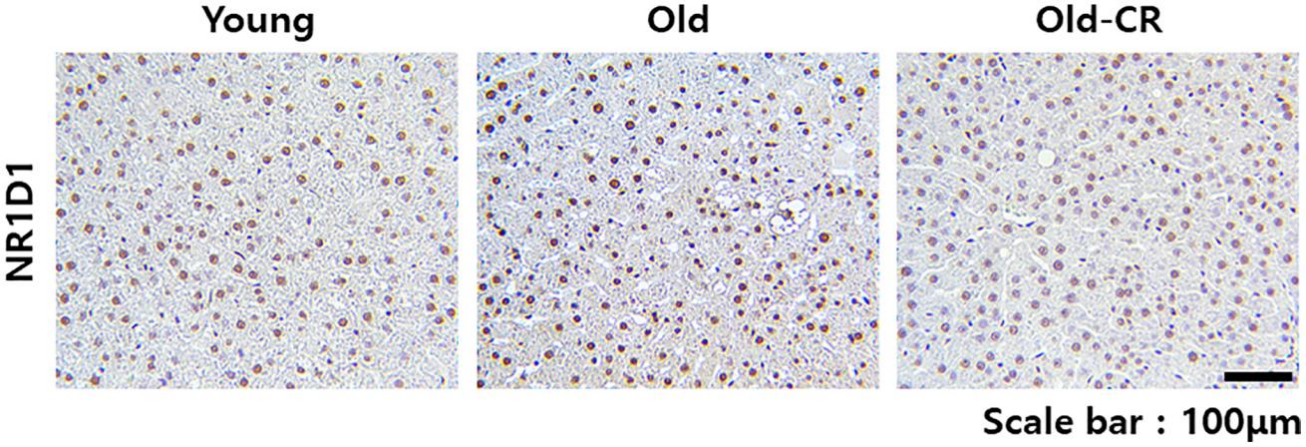
**Immunohistochemical analysis of Nr1d1 expression in rat liver tissues during aging and CR.** Nr1d1 protein expression is elevated in liver sections from aged mice compared to young controls, while CR intervention significantly attenuated this age-associated increase. Representative images of Nr1d1 staining of liver sections from the Young (left), Old (center), and Old-CR groups (right). Scale bar = 100 μm.

Western blotting detected Nr1d1 and Pparα levels (Figure 8). Nr1d1 protein levels were higher in the Old group than Young control but significantly downregulated in the Old-CR compared to Old. Conversely, Pparα protein levels were lower in Old than Young but increased with CR treatment. Immunohistochemistry assessed the Nr1d1 expression and localization in rat liver tissues (Figure 9). The Old group showed greater Nr1d1 intensity than Young, while intensity was significantly diminished in Old-CR compared to Old. These results suggest that the Nr1d1 and Pparα protein expression are affected by aging and CR with opposing trends, highlighting their potential as aging modulators.

### Effect of NR1D1 overexpression on PPARα binding to peroxisome proliferator response elements (PPRE) and the expression of circadian- and lipid metabolism-related genes

**Figure 10:**
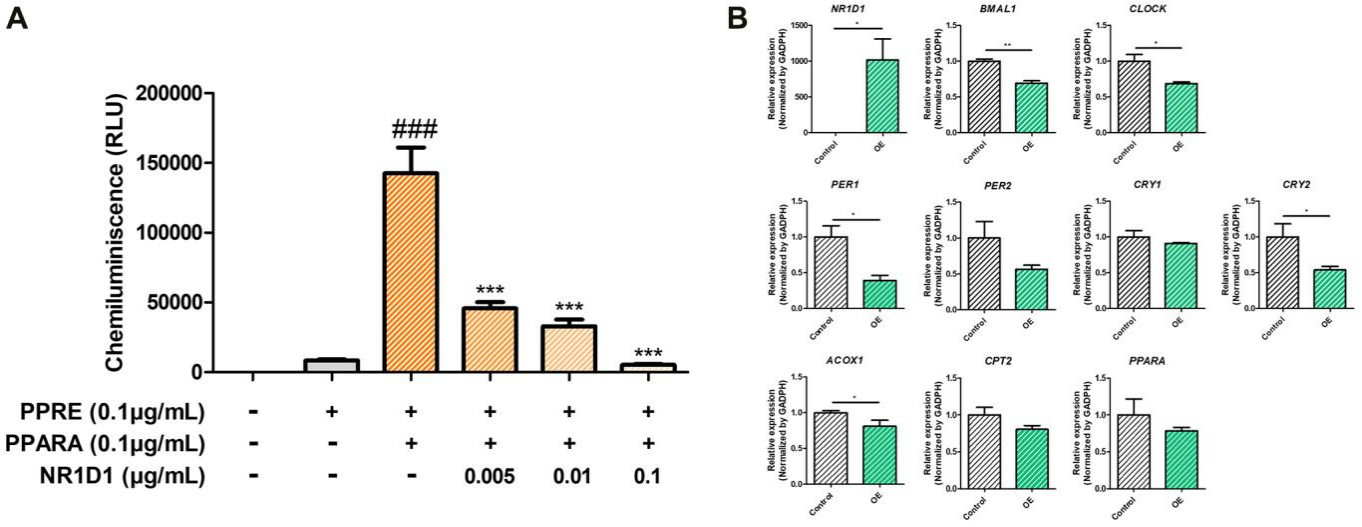
Effects of *NR1D1* overexpression on (**A**) the PPRE binding activity of PPARα and (**B**) expression changes of circadian and lipid metabolic genes (*n* = 6 per group). HepG2 cells were transfected with pcDNA or the *NR1D1* plasmid. The luciferase reporter assay measures the binding of PPARα to the PPRE element with varying amounts of *NR1D1* expression vectors. PPRE binding of PPARα decreases in a NR1D1 dose-dependent manner. Furthermore, the circadian gene *BMAL1, CLOCK, PER1/2,* and *CRY1/2* and lipid metabolic genes *ACOX1*, *CPT2,* and *PPARA* are decreased with *Nr1d1* overexpression. Data are presented as mean ± SEM. ^###, ***^p < 0.001 between two groups. OE; overexpression.

To investigate the relationship between NR1D1 and PPARα, we measured PPARα binding to PPRE in HepG2 cells using luciferase assay (Figure 10A). PPARα binding to PPRE decreased significantly in a dose-dependent manner with NR1D1 administration. To examine increased NR1D1 effects on gene expression during aging, we measured circadian rhythm and lipid metabolism gene expression in HepG2 cells overexpressing *NR1D1* (Figure 10B). Results showed decreased expression of *BMAL1, CLOCK, PER1, PER2, CRY1,* and *CRY2* in *NR1D1*-overexpressing cells and reduced expression of fatty acid oxidation genes (*ACOX1*, *CPT2, PPARA*). These results suggest that NR1D1 competitively binds to promoters, inhibiting PPARα binding to PPRE and reducing fatty acid oxidation gene expression.

## DISCUSSION

In this study, we identified key regulatory genes in the liver during aging and CR through transcriptomic and biological analyses. Transcriptomic analysis revealed changes in metabolic and circadian-related gene expression during aging and CR, with lipid metabolism showing opposite expression patterns. Notable changes in circadian gene expression were confirmed, particularly *Nr1d1*, which increased during aging and decreased with CR. PPI analysis predicted that *Nr1d1* association with lipid metabolism genes, including *Ppara*. We demonstrated that NR1D1 competitively binds to the PPRE against PPARα in human cells, reducing fatty acid oxidation gene transcription. These findings suggest that Nr1d1 overexpression during aging interferes with Pparα, leading to hepatic lipid accumulation, which was alleviated by CR.

Our study confirmed significant changes in genes associated with lipid metabolism and circadian rhythm during aging and CR. These findings align with prior literature demonstrating that circadian rhythms and lipid metabolism undergo substantial alterations during hepatic aging, and directly linked to age-related metabolic diseases [29]. The complex interplay between metabolic, hormonal, and cellular mechanisms contributing to age-associated lipid accumulation has been well-documented [43, 44]. Aging modifies the circadian transcriptome of the liver, resulting in tissue-specific alterations in circadian gene expression patterns [25, 45].

CR, a well-known anti-aging strategy, positively affects circadian rhythm gene expression, helping synchronize circadian rhythms and protect against desynchronization that negatively affects longevity [46]. CR also enhances protein modifications such as acetylation, suggesting a pathway to slow aging by modulating liver metabolism [25]. These findings are consistent with our data indicating significant alterations in circadian rhythm and metabolism-related gene expression during hepatic aging and CR.

We confirmed that *Nr1d1* is upregulated during aging but downregulated by CR. Our previous study demonstrated *Nr1d1* overexpression during hepatic aging [41]. Other studies reported that *Nr1d1* expression is associated with aging and lipid metabolism. For example, reducing *Nr1d1* levels in aged mouse heart cells promotes growth and reduces cell death, offering a strategy to mitigate heart aging [47]. CR changes transcriptomic levels of longevity and circadian-related genes, including *Nr1d1*, thereby mitigating circadian disruption [48–51].

*Nr1d1* plays a complex, context-dependent role in lipid metabolism regulation. While *Nr1d1* suppression leads to increased hepatic lipid accumulation, particularly with high dietary fat intake [52, 53], other studies reported that NR1D1 positively regulates lipogenic genes such as *SREBP-1c* in hepG2 cells [54]. These seemingly contradictory findings likely reflect the multifaceted nature of Nr1d1 function, which varies depending on metabolic state, tissue context, and experimental conditions. Our data revealed that in the aging context, *Nr1d1* overexpression disrupts the balance of lipid homeostasis by interfering with Pparα-mediated fatty acid oxidation, leading to lipid accumulation. This is consistent with studies showing that circadian clock proteins regulated by Nr1d1, including Bmal1, Clock, and cryptochromes, significantly regulate ketogenesis by interacting with Pparα in mouse models [55].

Our findings suggest that during aging, elevated Nr1d1 expression shifts this balance toward lipid accumulation by competitively inhibiting Pparα function, while CR restores metabolic balance by normalizing Nr1d1 levels. Although previous research on *Nr1d1* overexpression has been limited, our study highlights a novel role for Nr1d1 in significantly influencing lipid metabolism. This expands the understanding of Nr1d1 functions beyond its established roles, emphasizing the importance of maintaining Nr1d1 homeostasis as a potential strategy for regulating circadian rhythms and metabolism. Further research is warranted to explore these mechanisms in greater detail.

Our study confirmed that both Pparα expression and activity decline during liver aging but are restored by CR. PPARs play crucial roles in lipid metabolism regulation and prevention of obesity and diabetes, which can modulate aging [56–58]. Pparα, a member of the PPAR family, plays a protective role against age-related lesions in vital organs, including the liver, kidneys, and heart, thereby mitigating aging progression [59, 60]. These studies indicate that age-related PPARα decreases can aggravate the aging effects, highlighting its role as an aging modulator. On the other hand, CR leads to metabolic adaptations where the body reduces lipogenesis and increases lipolysis and ketogenesis by activating Pparα [9, 61]. Considering the critical role of Pparα as a metabolic regulator, comprehensive understanding of its targets and regulatory mechanisms could provide a robust foundation for addressing metabolic decline during aging.

Pparα is influenced by circadian genes and reciprocally regulates these genes while modulating lipid metabolism. Bmal1, in cooperation with Clock, activates *Ppara* expression by binding to E-box elements and acts as an upstream regulator of *Ppara* gene influencing various metabolic processes [62, 63]. Conversely, PPARα directly regulates core circadian rhythm components, such as BMAL1 and NR1D1, illustrating their significant role in metabolism and daily bodily cycles [64]. These findings support our study’s assertion of Pparα-Nr1d1 interaction during aging by demonstrating that Pparα functions as a regulator of circadian rhythm components, including Nr1d1.

We confirmed that NR1D1 competitively binds to the PPRE and inhibits the binding of PPARα to PPRE in hepG2 cells. NR1D1 modulates gene expression by antagonizing the binding of PPARα to the PPRE, illustrating a convergence of NR1D1 and PPAR signaling pathways in the transcriptional regulation affecting lipid metabolism, including β-oxidation [65–67]. In mouse models, Nr1d1 represses downstream circadian genes like *Bmal1* and *Clock,* while Pparα regulates the circadian clock by modulating Bmal1 [63, 68]. Bmal1/Clock reciprocally transactivates PPAR target genes via PPRE, indicating bidirectional influence of circadian and metabolic regulators [69]. These studies correspond with our findings that Nr1d1 plays a crucial role in modulating lipid metabolism by regulating fatty acid oxidation-related gene expression, which are principal Pparα targets.

**Figure 11:**
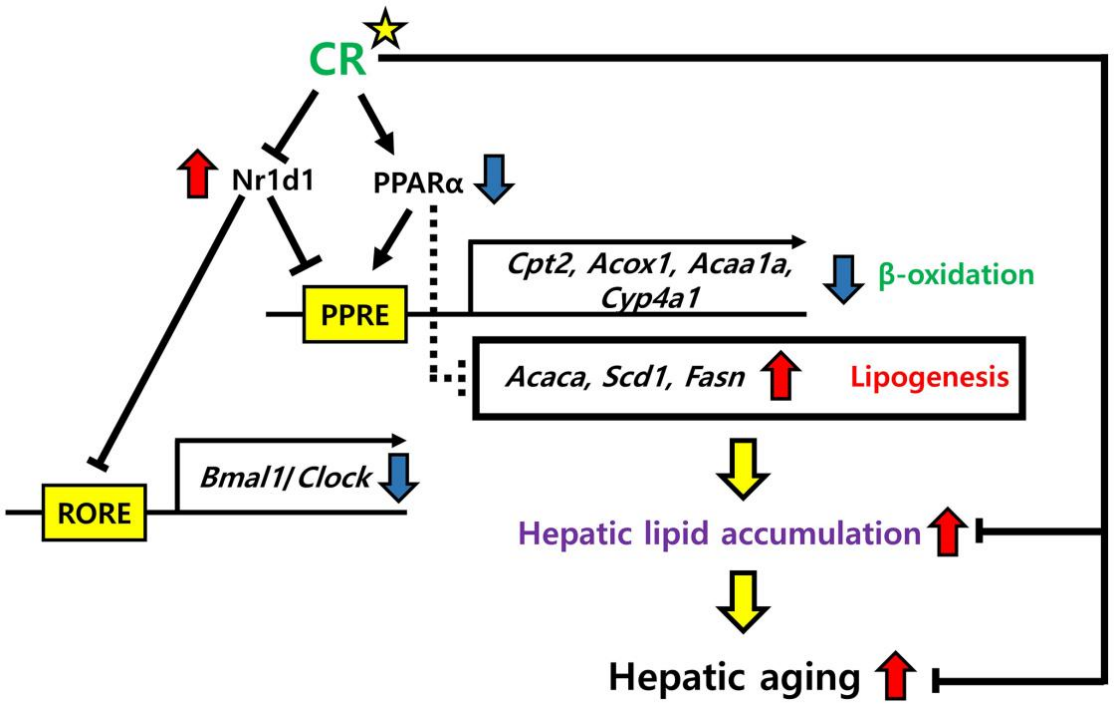
**Possible mechanisms of NR1D1 and PPARα modulation of hepatic lipid metabolism in aging and CR.** NR1D1 and PPARα competitively bind to PPRE. Upregulation of NR1D1 during aging represses the expression of genes involved in β-oxidation, such as *Cpt2*, *Acox1*, *Acaa1a*, and *Cyp4a1* and further induces the expression of lipogenic genes, such as *Acaca*, *Scd1*, and *Fasn*. These transcriptomic changes lead to hepatic lipid accumulation, which aggravates hepatic aging. However, CR may protect against the deterioration of lipid metabolism in the liver by modulating NR1D1 and PPARα.

In conclusion, our findings reveal that the co-regulation of Nr1d1 and Pparα plays a significant role in modulating hepatic lipid accumulation during aging, while CR may protect against this process, offering new potential strategies to mitigate the age-related metabolic decline with liver aging (Figure 11).

## MATERIALS AND METHODS

### Animals

Young male Sprague Dawley (SD) rats (Young group; 6 months old), old male SD rats (Old group; 19 months old), and old male rats treated with caloric restriction (Old-CR group; 19 months old) were purchased from Samtako (Osan, Gyeonggi-do, Korea). The CR protocol followed established methodologies where old-CR rats received 60% of the average daily food intake measured after 1-week stabilization, maintained for 4 weeks. Young and old rats were provided ad libitum water and a normal chow diet (20% protein, 4.5% fat, 6% fibre, 7% ash, 0.5% calcium, 1% phosphorus) [14, 42]. Rats were kept at 23 ± 2°C, with 60 ± 5% humidity and a 12-h light/dark cycle. Tissues were frozen in liquid nitrogen for analysis. All experiments were approved by the Pusan National University Institutional Animal Care and Use Committee (approval number PNU-2015-1044).

### RNA-Seq

Total RNA was extracted from liver samples using RiboEx reagent (GeneAll Biotechnology, Seoul, Korea). Equal quantities of RNA from each group (*n* = 5) were pooled for RNA-Seq analysis. cDNA libraries were prepared and sequenced using the MGI-T7 platform (MGI Tech Co., Shenzhen, China) with MGIEasy RNA Directional Library Prep Set. Adapter sequences were removed using Cutadapt (version 2.9) and quality filtered using Trimmomatic (version 0.39) [70, 71]. Reads shorter than 36 bp were excluded from analysis. Reads were aligned to the human reference genome (hg38) and Ensembl (version 102) using STAR (version 2.7.3a). STAR and RSEM (version 1.3.1) were used for alignment and quantification with default parameters [72, 73].

### Differential expression analysis

Gene count data were normalized using the DESeq2 normalization methods. Differential expression analysis was performed using DESeq2 (version 1.30.1) [74]. Differentially expressed genes (DEGs) were identified using *p*-values < 0.05 and absolute fold changes ≥1.5 (Supplementary Table 1). Volcano plots were generated using VolcaNoseR web interface [75].

### Gene ontology (GO) and pathway enrichment analyses

GO enrichment analysis examined biological functions, and KEGG pathway analysis investigated DEG-related signaling pathways. Both analyses were conducted using DAVID with statistical threshold of *p* < 0.05. Results are listed in Supplementary Table 2.

### qRT-PCR

Primers were synthesized by Bioneer (Daejeon, Korea). Total RNA was isolated from rat liver tissues (20 mg) and *NR1D1*-overexpressing HepG2 cells using the RNeasy Mini Kit (Qiagen, Hilden, Germany) (*n* = 6 per group) and reverse-transcribed using cDNA synthesis kit (GenDEPOT, Baker, TX, USA). qRT-PCR was performed using SYBR Green (Bioneer, Daejeon, Korea) and CFX Connect System (Bio-Rad, Hercules, CA, USA). Unpaired Student’s t-test was used for two-group comparisons (*NR1D1* overexpression vs. Control). When comparing multiple groups simultaneously, one-way analysis of variance (ANOVA) was performed followed by Tukey’s multiple comparison post-hoc test to control for family-wise error rate (Young vs. Old vs. Old-CR). Primer sequences are listed in Supplementary Table 4.

### Serum biochemical analysis

Serum samples were prepared by centrifugation (4°C and 2,000×g for 15 min) after euthanasia. TG levels were measured using serum kits (Bioassay Systems, Hayward, CA, USA). Serum TG levels were compared between groups using one-way ANOVA followed by Tukey’s post-hoc test.

### PPI network analysis

Mixed PPI database was constructed based on the interactions between five databases: HPRD, BioGRID, IntAct, MINT, and STRING [76–80]. Interactions with highest confidence (0.900) were selected from STRING, with all interactions extracted from the other databases. PPI network was constructed to identify hub molecules and examine DEG interactions. Cytoscape (version 3.10.1) visualized networks and calculated topological parameters including degree and betweenness centrality [81].

### Random Walk with Restart (RWR) algorithm on the PPI network

RWR algorithm was applied for node ranking within the PPI network to identify pivotal genes near seed genes [82, 83]. The mathematical underpinning of RWR involves the utilization of a transition matrix (M) and probability vectors, where P signifies the vector denoting node probabilities. The updated equation is expressed, as follows:


$$P\;(t+1)=(1-\alpha )\;.\;M.\;P(t)+\alpha \;.\;P_{\text{init}}$$

Here, α represents the restart probability (damping factor), P(t) denotes the probability vector at iteration t, and P_init_ is the initial probability vector. RWR computations used 0.8 restart probability with R package RandomWalkRestartMH [84]. Results are presented in Supplementary Table 6.

### Western blotting

Total protein was extracted from rat liver tissues and boiled for 5 min in loading buffer containing 0.2% bromophenol blue, 125 mM Tris-HCl, 10% 2-mercaptoethanol, and 4% SDS (pH 6.8) (*n* = 6 per group). Equal amounts of protein (8–10 μg) were separated by SDS-PAGE using 10% gels and transferred to PVDF membranes at 25 V for 10 minutes using a semidry transfer. Membranes were blocked in 5% nonfat milk for 2 h, then immunoblotted overnight with primary antibodies (1:1,000) at 4°C. After washing, the membranes were incubated with horseradish peroxidase (HRP)-conjugated secondary antibodies (1:10,000) for 1 h at room temperature. Protein detection was performed using enhanced chemiluminescence, and molecular weights were determined using a wide range of protein markers. Immunoblots were visualized using a chemiluminescent HRP substrate (Davinchchemi CAS-400) and analyzed using the ImageJ Software. The detailed procedure has been described previously [41]. Antibodies against NR1D1 (sc-100910), PPARα (sc-398394), and α-tubulin (sc-5286) were purchased from Santa Cruz (Santa Cruz Biotechnology, Santa Cruz, CA, USA). Secondary antibodies (GTX213110-01 and GTX213111-01) were purchased from GeneTex (Irvine, CA, USA). Protein levels were compared between groups using one-way ANOVA followed by Tukey's post-hoc test.

### Immunohistochemistry

Paraffin-embedded sections were deparaffinized of using xylene, followed by graded ethanol series (starting at 100% and ending at 70%) and rehydration. Antigen retrieval was performed using sodium citrate buffer (pH 6.0). Specimens were then incubated with Nr1d1 primary antibodies (Santa Cruz, sc-100910, 1:200) at 4°C overnight in a humidity-controlled environment. After PBS washing, sections were incubated with biotinylated secondary antibodies (1:10,000; VectorLabs) for 30 min at ambient temperature. Diaminobenzidine substrate with hematoxylin counterstaining was used for visualization. Microscopic examinations were performed using a Motic AE30/31 inverted microscope.

### Cell culture

HepG2 cells were purchased from American Type Culture Collection and maintained in Dulbecco’s Modified Eagle Medium (Welgene; LM001-11) containing 10% fetal bovine serum (Gibco; S001-01), 100 U/mL penicillin, and 100 μg/mL streptomycin (Hyclone; SV30010) at 37°C in 5% CO_2_. Cells were cultured in sterile plastic plates (SPL, 20100).

### Cell transfection for NR1D1 overexpression

To observe the change in mRNA expression with *NR1D1* overexpression, HepG2 cells were transfected with pcDNA or the NR1D1 plasmid using Lipofectamine 3000 (Invitrogen; L3000015), according to the manufacturer’s instructions. The *NR1D1* plasmid was kindly provided by Dr. Mi-Ock Lee (Seoul National University, Seoul, Republic of Korea).

### Luciferase assay

To observe the binding of PPARα or NR1D1 to PPRE, HepG2 cells (1.5 × 10^4^ cells/well) were grown in a 96-well plate in DMEM supplemented with 10% FBS. The PPRE-X3-TK-LUC plasmid (0.1 μg) purchased from BioCat company (Heidelberg, Land Baden-Württemberg, Germany), and *PPARA* and the *NR1D1* plasmid were transfected using Lipofectamine 3000 (0.1 μL) and P3000 (0.2 μL) complexes in Opti-MEM, according to the manufacturer’s instructions. An empty pcDNA vector was added to ensure that equal amounts of plasmid DNA were used per transfection. Luciferase activity was measured using a ONE-Glo Luciferase Assay System (Promega, Madison, WI, USA) and a luminescence plate reader (Berthold Technologies GmbH & Co., Bad Wildbad, Germany). Data were analyzed using one-way ANOVA with Tukey’s post-hoc test for multiple comparisons.

### Statistical analysis

Sample sizes (*n* = 6 per group for animal studies, *n* = 3 for cell culture experiments) were determined based on our previous studies to detect meaningful biological differences [27, 85]. All statistical analyses were performed using the GraphPad Prism software (version 5; GraphPad Software, La Jolla, CA, USA). Data are presented as mean ± standard error of the mean (SEM). Statistical significance was set at *p* < 0.05 for all analyses.

## Supplementary Materials

Supplementary Figure 1

Supplementary Table 1

Supplementary Table 2

Supplementary Table 3

Supplementary Table 4

Supplementary Table 5

Supplementary Table 6
