# Supplementary material for: Co-regulation of Nr1d1 and Pparα in age-related changes of lipid metabolism and its modulation by calorie restriction
Source: Aging (Albany NY). 2025 Jul 28;17(7):1810–33. doi: 10.18632/aging.206289 (PMC12339034; doi:10.18632/aging.206289)
Supplement: Supplementary Figure 1 [file aging-17-206289-s001.pdf]

SUPPLEMENTARY FIGURE

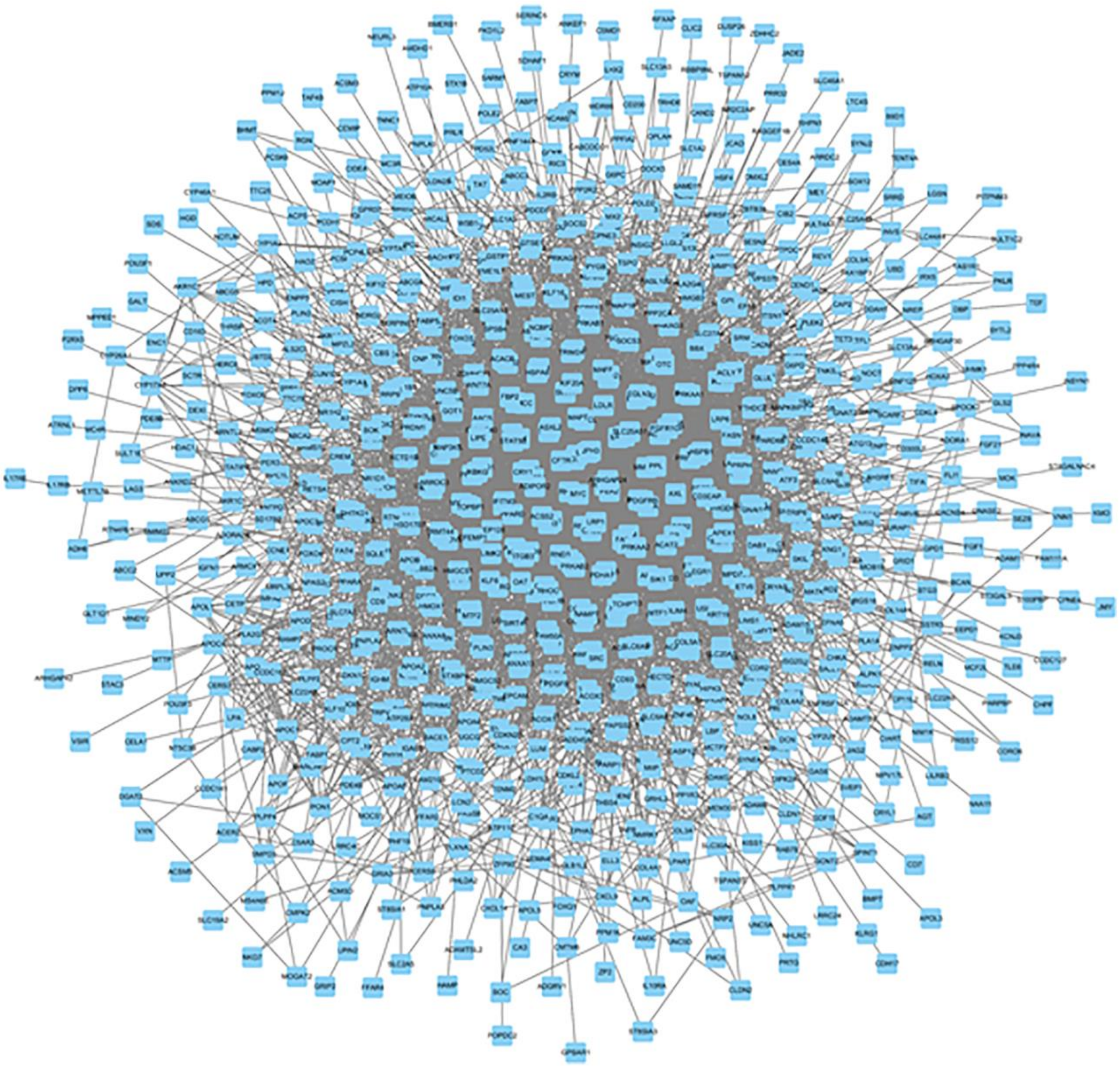

Supplementary Figure 1. PPI network constructed using genes related to the circadian rhythm and lipid metabolism and age-affected DEGs.
