# Supplementary material for: Co-regulation of Nr1d1 and Pparα in age-related changes of lipid metabolism and its modulation by calorie restriction
Source: Aging (Albany NY). 2025 Jul 28;17(7):1810–33. doi: 10.18632/aging.206289 (PMC12339034; doi:10.18632/aging.206289)
Supplement: Supplementary Table 4 [file aging-17-206289-s005.pdf]

SUPPLEMENTARY TABLE

Supplementary Table 4. Primer sequences for RT-qPCR analysis.

Species	Gene	Forward (5′–3′)	Reverse (3′–5′)
Rat	*Acaa1a*	CCTGATTCCTATGGGGATAACC	GTTGTCACAGGTACGATCTCAG
Rat	*Acaca*	GGCACTCTGATCTGGTCACG	GCTCCGCACAGATTCTTCAA
Rat	*Bmal1*	TATCACACTACGAAGTCGATGG	CGGCAATCATTCGACCTATTTT
Rat	*Clock*	GAGGTCATCCTTCAGTAGTCAG	TGAAAGCTGAAACTGTGACATG
Rat	*Cpt1a*	AAGCTGTGGCCTTCCAGTTC	GGATGAAATCACACCCACCA
Rat	*Cry1*	CCTTGATGCCAATCTACGAAAG	AAAGGCTCAGAATCGTACTCAA
Rat	*Cry2*	AAAACCACATGGGGAATAGACT	GTTTCTTTCACCAGTTCAGACC
Rat	*Cyp4a1*	CAAGGTGACAAAGAACTACAGC	CAAGGAGCTAGCAATCTGTAGA
Rat	*Fasn*	GCTGGGACACATGTGATGGT	AGTGAGTGTACGGGAGGGCT
Rat	*Nr1d1*	AATGCCAATCATGCATCAGGTA	ATTCAGTGCTTCATTATGACGC
Rat	*Nr1d2*	GAATTCGGTTGTACCACAAGAC	AGGTAAAGACGCTTTATGGACA
Rat	*Per1*	AACATTCCTAACACAACCAAGC	CTGCTGACGACGTATCTTTCTT
Rat	*Per2*	CTCTGACACATCCCAGTCTAG	GTCCCTGGTGTGGATACTATTC
Rat	*Ppara*	ACGGCGTTGAAAACAAGGAG	TTGGCAAATTCCGTGAGCTC
Rat	*Ppard*	CATTGTCAACAAAGACGGACTG	ATTGAACTTGACAGCAAACTCG
Rat	*Rora*	CAATATACCCAGACATTGTGCG	ACTCCAGATGTTCTAGAAGTGC
Rat	*Rorc*	GTGGAGCATCTGCAATACAATT	CAATGTCAGTGCTGAAGAGTTC
Human	*NR1D1*	TGAATGGCATGGTGTTACTG	ACTTCTTGAAGCGACATTGC
Human	*BMAL1*	CTGTGCTAAGGATGGCTGTT	GCTCCTTGACTTTGGCAATA
Human	*CLOCK*	GGCAAAATGTCATGAGCACTTA	AGCCCTAACTTCTGCATAACTT
Human	*PER1*	CAGCTTTTTATTGAGTCTCGGG	CAGTTGATCTGCTGGTAGGAG
Human	*PER2*	CTTATTCACTGCCCGTGTTTC	GGAAGGAATAACTGGGTAGCAT
Human	*CRY1*	GACGCAGCTATTAAGAAACTGG	TTTGCTGATGAGAGTCTGGAAT
Human	*CRY2*	CTATGAGAGACCCCGAATGAAC	CCGCTTCACCTTTTTATACAGG
Human	*ACOX1*	AGGTCACAGCTGTCCAACCA	TTACCCAGCCCTGGCTTAAT
Human	*CPT2*	GGTCCAGGTAGAGCTCAGGC	GTGCTCTGAGGCCTTTGTCA
Human	*PPARA*	TCGGCGAGGATAGTTCTGGAAG	GACCACAGGATAAGTCACCGAG
